# Investigation of the Incompatibilities of Cement and Superplasticizers and Their Influence on the Rheological Behavior

**DOI:** 10.3390/ma13040977

**Published:** 2020-02-21

**Authors:** Ursula Pott, Cordula Jakob, Daniel Jansen, Jürgen Neubauer, Dietmar Stephan

**Affiliations:** 1 Department of civil engineering, Building Materials and Construction Chemistry, Technische Universität Berlin, 13355 Berlin, Germany; u.pott@tu-berlin.de; 2 Department of Geography and Geosciences, GeoZentrum Nordbayern, Friedrich-Alexander-University, 91054 Erlangen, Germany; cordula.jakob@fau.de (C.J.); daniel.jansen@fau.de (D.J.); juergen.neubauer@fau.de (J.N.)

**Keywords:** rheology, incompatibility of superplasticizer, hydration, spread flow test, penetration, in-situ XRD, calorimetry, ultrasound, SEM

## Abstract

The rheological behavior of cement paste and the improvement of its flowability takes center stage in many research projects. An improved flowability can be achieved by the addition of superplasticizers (SP), such as polycarboxylate ethers (PCE). In order to be able to use these PCEs effectively and in a variety of ways and to make them resistant to changes in the environment, it is crucial to understand the influence of SPs on cement hydration. For that reason, the topic of this paper was the incompatibility of a specific SP and an ordinary Portland cement (OPC). The incompatible behavior was analyzed using rheological tests, such as the spread flow test and penetration test, and the behavior was compared by means of an ultrasound technique and explained by the phase content measured by in-situ X-ray diffraction (XRD) the heat evolution measured by calorimetry, and scanning electron microscope (SEM) images. We showed that the addition of the SP in a high dosage led to a prevention of the passivation of the most reactive and aluminum-containing clinker phases, aluminate and brownmillerite. This induced the aluminate reaction to take place in the initial period and led to an immediate stiffening of the cement paste and, therefore, to the complete loss of workability. The results showed that in addition to the ettringite, which began to form directly after water addition, hemicarbonate precipitated. The fast stiffening of the paste could be prevented by delayed addition of the SP or by additional gypsum. This fast stiffening was not desirable for SPs, but in other fields, for example, 3D printing, this undesirable interaction could be used to improve the properties of printable mortar.

## 1. Introduction

The flow behavior and the rheological properties (viscosity and yield stress) of fresh cement paste are of particular importance for filling formwork, for pumping fresh concrete, and for the new technology of 3D printing. The rheological behavior can be improved by the use of so-called superplasticizers (SP). For the correct use of SPs, it is essential to understand the chemical processes during the hydration of cement on the one hand, and the influence of SP on the hydration process itself on the other hand.

The hydration process can be separated into the dissolution of several phases and the precipitation of hydrate phases [1]. For early hydration, the most important phases in Portland cement are alite (C_3_S) and aluminum-containing phases, such as aluminate (C_3_A) and brownmillerite (ferrite or C_4_AF) [2,3]. C_3_S is the phase with the highest share of ordinary Portland cement (OPC). The proportion is 50 wt% to 80 wt% [4]. C_3_A is the most reactive phase, with a proportion of up to 15 wt% [5]. The hydration process can be separated into the silicate and aluminate reaction, as shown in the following equations:

Silicate reaction:

alite + water → C-S-H + portlandite (1)

Aluminate reaction:

aluminate phases + calcium sulfate + water → ettringite (2)

However, it is known that both processes take place simultaneously during cement hydration. During the silicate reaction, C_3_S dissolves, and low crystalline C-S-H phases (calcium-silicate-hydrate phases) and crystalline portlandite (Ca(OH)_2_) are formed. The C-S-H phases are responsible for the setting process and the rheological behavior of the cement paste at a later stage [6]. During the reaction of C_3_A, ettringite (AFt) is formed through the reaction of C_3_A, calcium sulfate, and water. The reaction rate of C_3_A in the absence of calcium sulfate is very high, which would lead to a very fast setting of the cement paste and a high heat evolution [7]. To ensure a sufficient workability time, calcium sulfate is added to the paste in order to control the reaction of C_3_A [3]. During the production of cement, the producer always has to adjust the right type and amount of calcium sulfate. If the amount of calcium sulfate is too low, ettringite and aluminate ferrite monosulfate (AFm) with a tabular morphology are formed during the very early hydration stage, resulting in insufficient workability of the fresh paste. This can lead to a so-called “flash set”. If the amount of dissolved calcium sulfate is sufficient, only ettringite with a more compact structure is formed, and the flowability is appropriate. However, if the initially dissolved amount of calcium sulfate is too high, gypsum (so-called secondary gypsum) forms in addition to ettringite [8]. This is caused by the added gypsum to the clinker, which can dehydrate to calcium sulfate hemihydrate (bassanite) during cement grinding. Bassanite is the highest soluble calcium sulfate and dissolves immediately upon water contact. If the amount of bassanite in the raw cement is too high, the precipitation of gypsum can lead to a so-called “false set”. In most cases, the gypsum is dissolved again during further mixing so that it has no influence on the rheological behavior [9]. Some researchers assume that in contrast to C_3_S, the reaction of C_3_A influences the workability and flowability at an early stage of cement hydration [2,10]. Other researchers, however, suggest that the formation of C-S-H mainly influences the early flowability [11]. The amount of sulfate in cement is not sufficient to transform all C_3_A into ettringite. However, ettringite is stable as long as enough dissolved calcium sulfate is available [12]. As soon as the amount of sulfate ions in the solution is too low, ettringite is converted to AFm phases.

The second aluminate-containing clinker phase is called brownmillerite or calcium aluminoferrite. The hydration of this phase is similar to C_3_A [13]. Without calcium sulfate, brownmillerite reacts quite fast, and iron-containing AFm-phases precipitate as well. With calcium sulfate, the hydration is retarded, and ettringite is formed.

**Figure 1:**
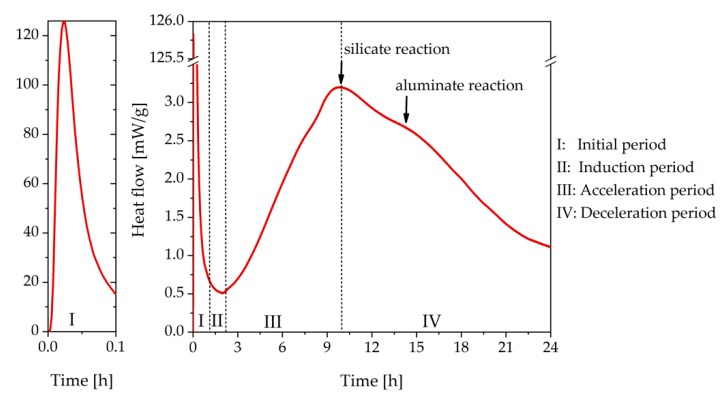
Heat flow curve of CEM I measured by calorimetry with internal mixing (water/cement, w/c = 0.36).

Cement hydration is an exothermic reaction with time-varying reaction rates. For that reason, the process can be divided into different stages based on the heat evolution measured by calorimetry (Figure 1) [2,3,14]. The first very rapid reaction is defined as the initial period (I). During this period, a large amount of heat can be detected. This heat evolution occurs mainly due to the first very rapid dissolution of C_3_A and calcium sulfate and the precipitation of ettringite [15]. After this fast reaction, the dissolution of C_3_A stops for a while. It is believed that the surface of C_3_A is passivated. The reason for that is still under debate. One explanation is that the formation of X-ray-amorphous ettringite leads to a diffusion barrier on the surface of C_3_A (and other clinker phases), which limits the transport of water and ions to the C_3_A surface [3,5]. However, this theory was recently discussed as somewhat questionable [16]. Other researchers found evidence that the adsorption of sulfate ions on the reactive surface caused the slowdown of the aluminate reaction [5,17]. Collepardi et al. suggested that in addition to sulfate ions, calcium ions were essential for the passivation as well [5,18,19]. However, during the period of no further C_3_A dissolution, the pre-dissolved C_3_A still reacts with calcium sulfate and forms ettringite.

Moreover, in the initial period, a small amount of C_3_S dissolves and reacts with water. The reason why the reaction of C_3_S slows down after this rapid reaction is also still under debate. Stein et al. and Bellmann et al. assumed that a thin metastable layer of C-S-H phases passivated the surface [20,21]. Other researchers thought that the deceleration was caused by a superficially hydroxylated layer [22]. Additionally, the so-called “dissolution theory” has been applied recently, suggesting that no passivating layer is needed in order to explain the hydration kinetic of C_3_S [16]. For more details about the different hypotheses, the reader is referred to [3]. The second period is called the induction period (II). During the induction period, almost no heat flow can be detected because only a slow reaction, namely the dissolution of sulfate and the precipitation of ettringite, takes place [2]. Once C_3_S starts to react and C-S-H phases and portlandite are formed, the acceleration period of the silicate reaction begins (III). During this time, the heat evolution increases rapidly. At that stage, the hardening process of the cement paste proceeds. After the first peak caused by the C_3_S reaction, the deceleration period (IV) of the silicate reaction begins, and a second peak appears, which is caused by the ongoing C_3_A dissolution initiated by the exhaustion of sulfate. Period III and IV are combined as the main period. In the last stage, the heat evolution decreases, and the curve flattens again [2]. 

The workability of the paste during hydration is mainly influenced by the chemical reactions taking place during the hydration process. In addition, the particle size distribution, the volume fraction of the solid, as well as the aqueous phase and attractive interparticle forces, influence the yield stress and have a significant impact on the rheological behavior [8,23]. Particle forces, such as van der Waals and electrostatic forces, lead to the flocculation of particles. The size and shape of the agglomerates influence the flowability of the paste. In general, the flocculation of particles in a cement paste leads to a decrease in workability.

Hence, one way to improve the flow behavior (workability) of a cement paste is the use of superplasticizers (SP). The most efficient and, therefore, most frequently used SPs are polycarboxylate ethers (PCE) [23]. These molecules generally have a comb-like structure and consist of a backbone with side chains [23,24]. PCEs consist of a polymeric backbone, consisting mainly of carboxylic acid groups and attached side chains of polyethers [23,25]. These backbones attach to the surface of the clinker particles due to the high charge introduced by the carboxylic groups, while the side chains tangle into the solution [23]. The liquefying effect of SPs is due to the deflocculation of the cement particles caused by two mechanisms, namely electrostatic repulsion and steric repulsive hindrance between the particles introduced by the adsorbed PCE molecules. The electrostatic repulsion is achieved by the same charge on the surfaces of the clinker particles provided by the adsorbed SPs, and the steric repulsive hindrance is generated by the hydrophilic side chains, which protrude into the solution (osmotic effect). In the case of PCEs, the steric repulsive hindrance is the dominating mechanism. Due to this steric repulsive hindrance, the attractive forces between the cement particles are reduced, and the clinker particles can move freely against each other [23,26]. This, in turn, improves the flowability of the cement paste. An often-considered disadvantageous side effect associated with the use of SPs is the retardation of hydration [27].

In general, the dosage of an SP can be divided into three stages. Below a critical dosage, the SP has no effect on the properties of the cement paste. The surface coverage of the cement particles by the SP is too low to prevent agglomeration. Beyond this critical dosage, the flow behavior improves with increasing dosage until saturation of the SP on the surfaces is achieved. At the saturation dosage, the surface is completely covered, and the yield stress reaches a minimum value [28]. Above this saturation dosage, further addition of SP does not improve the flow behavior [23].

Moreover, it is known that the effectiveness of SPs can be enhanced by delayed addition [23,29,30]. In contrast to a direct addition, together with the mixing water, a delayed addition leads to an increased flowability but simultaneously to more pronounced retardation [23]. 

During cement hydration, the first hours are of interest for the evolution of the rheological behavior, which must be adjusted for pumpable concrete or concrete for 3D printing, for example. Rheological tests can be performed until the material is too stiff for the tests. For an OPC, this level is reached during the acceleration period [9]. There are many papers dealing with the rheology of cement pastes and with the enhanced flowability through the addition of SPs. Nevertheless, there are fewer papers dealing with the problem when the addition of an SP leads to a loss of fluidity instead of an improvement. Marchon et al. tried to explain this phenomenon. They found that in some cases, the C_3_A reaction could be accelerated so that this reaction takes place before the silicate reaction. Moreover, they mentioned that this behavior could only be observed at very high dosages, with highly charged molecules, and with direct addition and not by delayed addition of the SP [24]. 

The aim of this paper was to analyze the incompatibility of one specific SP with an OPC of the type CEM I 42.5 R. This incompatibility occurred if the SP was overdosed and directly added. We analyzed the flow behavior of the cement paste with a spread flow test, a penetration test, and ultrasound measurements and compared the results with the heat evolution (calorimetry), the phase formation (in-situ X-ray diffraction (XRD)), and scanning electron microscope (SEM) images. We found out what caused the incompatibility and how it could be prevented. We investigated whether the behavior could be improved by adding additional calcium sulfate (gypsum) and what effect a delayed addition of the SP had. Since the aluminate reaction played a key role in understanding the changes in rheological behavior between the different systems studied in our manuscript, we concentrated mainly on the hydration of the aluminum-containing phases during OPC hydration in this paper.

## 2. Materials and Methods

### 2.1. Materials

For the investigations performed, an ordinary Portland cement (OPC, CEM I 42.5 R, HeidelbergCement, Heidelberg, Germany) was used. Its chemical and mineralogical composition is given in Table 1. The specific surface measured by Blaine was 3699 ± 23 cm²/g, and the value for loss on ignition was 1.9 wt%. Deionized water was used for all experiments. For more information about the cement, the reader is referred to [31].

The used SP, provided by BASF, was designed for ready-mixed concrete. These SPs should lead to good workability of the concrete for several hours [23]. The chemical data are given in Table 2.

### 2.2. Sample Preparation

Samples for the penetration test, in-situ XRD, ultrasound, spread flow test, and SEM images were produced by mixing 650 g cement with water using a water/cement ratio (w/c ratio) of 0.36 at 20.0 ± 0.3 °C and relative humidity of 65%. The paste was prepared with a stand mixer (Kitchen Aid, Type 5K45, Benton Habor, MI, USA) Two different mixing procedures were used—one for the direct addition of the SP and another one for the delayed addition. For the direct addition, the water was mixed with the SP and added to the cement paste. The mixing procedure started at level 1 (58 rounds per minute (rpm)) for 15 s. After that, the speed was increased to level 4 (ca. 125 rpm) for 45 s. This was followed by a 30 s break to rid the edges of the bowl of cement paste. The last step involved mixing at level 4 again for 30 s. The whole mixing process took 2 min. For the delayed addition, 90 wt% of the water was added to the cement, and the cement paste was mixed at level 1 for 15 s and at level 4 for 45 s. After a break of 30 s, the remaining 10 wt% of the water was added together with the SP to the cement paste. Finally, the cement paste was mixed for 30 s at level 4. Care was taken to obtain a homogeneous paste from the mixing procedure. For calorimetry, the paste was mixed in a special device named InMixEr (Section 2.6) [32] at 860 rpm for 1 min inside the calorimeter so that the first heat evolution could be detected without artifacts.

### 2.3. Spread Flow Test

The spread flow test is a commonly used method to analyze the flow behavior of a paste and to determine the yield stress. The procedure is described in the standard DIN EN 1015-3 [33]. For the test, a mold was filled with the material. Seven and a half minutes after the addition of water, the mold was lifted, and the material flowed. It is known that the material stops flowing as soon as the shear stress in the tested paste is lower than the yield stress of the paste [34]. As a result of the test, the spread diameter could be measured, which represented the so-called slump flow. Roussel et al. established an equation (3) to determine the yield stress *τ_0,_* depending on the density *ρ* (kg/m³), the gravity *g* (m/s²), the sample volume *Ω* (m³), and the radius *R* (m) without shocks [34,35]. This equation is suitable for pastes with a slump flow diameter > 20 cm [36].

(3) $$\tau_{0}=\;\frac{225{\;\rho \;g\;\Omega }^{2}}{128{\;\pi }^{2}{\;R}^{5}}$$

Cement paste showed a shear thinning behavior. That means that the rheological values, such as yield stress and viscosity, varied depending on the applied shear. At lower shear conditions, yield stress and viscosity were higher than with a higher shear. This must be considered when comparing rheological values from various studies. For the spread flow test, a less sheared paste was tested.

### 2.4. Penetration Test with a Multipurpose Incremental Loading Device (MILD)

The MILD is a custom-made penetration test device. Interested readers can find more information about the device elsewhere [37]. During the measurement, the cement paste was penetrated with a speed of 1.2 µm/s. The penetration geometry used was a sphere with a diameter of 10 mm. During the measurement, the force needed to penetrate the cement paste was detected. This force increased with the ongoing hydration process of the cement. The measured force was mainly due to the yield stress of the sample [38]. Thus, this penetration test could be seen as a rheological testing method. Due to the low speed during the test, the paste was almost unsheared.

### 2.5. In-Situ X-ray Diffraction (XRD)

The phase evolution was determined by in-situ XRD measurements. The temperature was kept constant at 20°C ± 0.2°C. To prevent water evaporation and CO_2_ intake, the sample was covered by a 7.5 µm thick Kapton® polyimide film (FLUXANA GmbH & Co. KG, Bedburg-Hau, Germany). Every 10 min, the diffraction patterns were recorded by a Bruker AXS D8 Advance diffractometer (Karlsruhe, Germany) with a Bragg-Brentano geometry from 7° to 55° 2θ and step width of 0.0236° 2θ. The XRD device has a Lynx-Eye detector, and Cu Kα radiation (Ni-filtrated) was used. The power generator operated at 40 mA and 40 kV. The measurement duration was 48 h. A Rietveld analysis was done with the software TOPAS 5.0 (Bruker AXS). Moreover, an external standard G-factor method [15] was used. Therefore, the weights in wt% were absolute values of all phases. They took into account both the significant amorphous content caused by the water added for hydration and not determined phases.

For the sample without PCE and the sample with direct addition of 0.7 wt% PCE, no bleeding occurred. For the other systems additionally, samples at different points in hydration time were stopped by solvent exchange and gently dried at room temperature. The results of XRD measurements of these samples were compared to the in-situ measurements. They showed good accordance; thus, the in-situ measurements were showed in this study.

### 2.6. Heat Flow Calorimetry

The heat evolution caused by the hydration of the paste was measured by a TAM air calorimeter (TA instruments, New Castle, PA, USA) at a constant temperature of 20°C ± 0.2°C. For the internal mixing, a custom-made InMixEr [32] device was used. The cement pastes were prepared by internal injection of the water and mixing at 860 rpm for one minute. It was checked whether the mixing process of the cement paste influenced the heat evolution during cement hydration. It could be seen that the mixing procedure did not influence the main period significantly. To be able to evaluate the first peak in the initial period, as this area is of great importance for this investigation, the samples for calorimetry were mixed internally. For the delayed addition, the cement was manually mixed with 90 wt% of the water for 20 s before the remaining water with SP was added. In accordance with the temporal resolution of the calorimeter, the data were corrected according to the Tian equation [39]:

(4) $$P_{c}\left(t\right)=P_{0}\left(t\right)+\;\tau \frac{dP}{dt}$$

*P*_c_ = corrected thermal power (W)

*P*_0_ = measured thermal power (W)

τ = time constant (s)

*t* = time (s)

### 2.7. Ultrasonic Measurements

Ultrasound is a nondestructive method for measuring the microstructural changes in the cement paste during hydration. Other researchers have already shown that this method is suitable for cement paste [29,38,40]. For this investigation, a device type IP8 produced by UltraTest GmbH was used. In this test, fresh cement paste was filled into a mold. A transmitter that generates ultrasonic waves with a frequency of 25 kHz was placed on one side of the mold, and on the opposite side of the mold, a receiver detected the ultrasonic waves after they passed through the sample. The distance between the transmitter and the receiver was fixed. The velocity of the ultrasonic wave could be determined by measuring the time needed for the waves passing through the sample and the distance between the transmitter and receiver. This velocity depended on the microstructure of the paste. Due to cement hydration, the microstructure changed, and the velocity increased. Thus, the ongoing hydration process could be observed.

### 2.8. Scanning Electron Microscope (SEM)

For SEM measurements, the hydration was stopped, and water replaced with isopropanol. The mixing ratio of cement paste and isopropanol was 1:3, and the isopropanol was replaced 3 times every 3 min to ensure a complete stop of hydration. The dried powder was analyzed with a scanning electron microscope (Zeiss GeminiSEM 500 NanoVP, Oberkochen, Germany). Before the measurement, the sample was covered by a 3 nm thick gold layer. For the pictures, a secondary electron (SE) detector in a high vacuum was used, and the electrons were accelerated in an electric field with a voltage of 15 kV. 

### 2.9. Thermodynamic Modeling

The thermodynamic modeling of the stable hydrate phase assemblages for the full hydration of CEM I was performed based on the recently published CEMDATA 18 database [41] using the GEMS-PSI software V3.5 [42,43]. The calculation was done for a temperature of 20 °C and a w/c ratio of 0.36. The chemical composition in Table 1 was used to calculate the thermodynamic equilibria. TiO_2_ was not considered.

## 3. Results

### 3.1. Cement Paste Without Superplasticizer

In this chapter, the system without SP was analyzed. Figure 1 shows the heat flow curve of the investigated OPC measured by calorimetry. This figure shows the initial (I), the induction (II), the acceleration (III), and the deceleration period (IV), as described in the introduction. The peak related to the silicate reaction occurred at about 10 h, and the peak related to the aluminate reaction at 15 h. 

**Figure 2:**
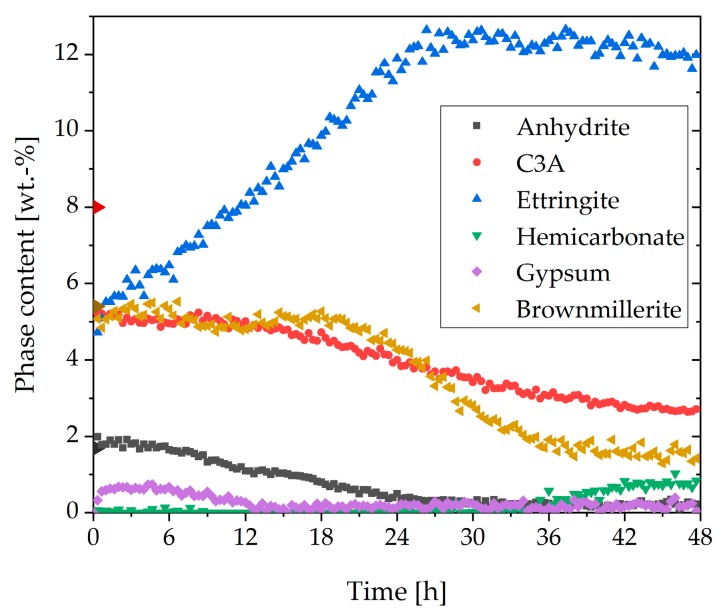
Phase development of the aluminum-containing phases of CEM I during 48 h after water addition (w/c = 0.36).

In Figure 2, the phase contents of anhydrite, C_3_A, ettringite, hemicarbonate, gypsum, and brownmillerite (w/c ratio = 0.36) are shown for the first 48 h of hydration. Bassanite was completely dissolved during the first 10 min and was, therefore, not shown. The phase content of C_2_S and C_3_S was not displayed because it was constant in the first two hours and, therefore, not important for this investigation. The initial content of anhydrite was 1.6 wt%. The amount steadily decreased until 25 h. The initial content of C_3_A was 8 wt%. During the initial reaction, 2.8 wt% was dissolved, and in the first minutes, nearly 5 wt% of ettringite was already formed. During the following 12 h, no further dissolution of C_3_A could be detected. The amount of ettringite increased constantly until 27 h to a maximum of 12.6 wt%, followed by a slight decrease. No significant initial dissolution of brownmillerite was observable. The dissolution of this phase started after about 21 h. The limiting factor for the ettringite formation was the available amount of sulfate in the dry cement. For that reason, the ettringite formation was almost finished after all sulfate carriers were entirely consumed. As could be seen in Figure 2, gypsum could be detected during the first hours of hydration, but it was not part of the dry cement. It precipitated during the hydration and was consumed for the ettringite formation later on. 

**Figure 3:**
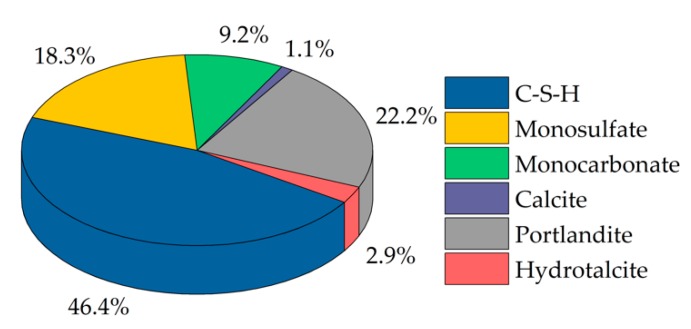
Composition of the hardened CEM I after an infinite duration of hydration (w/c = 0.36).

Figure 3 represents the thermodynamic stable hydrate phase assemblage of the Portland cement used, assuming complete hydration. The diagram clarified that ettringite was just a metastable phase in the paste since it was not stable after complete hydration. The formation of ettringite in the OPC was only due to kinetics. After complete hydration, C-S-H phases had the largest share (46.4 wt%). The second-largest share was portlandite (22.2 wt%). Monosulphate was represented with a share of 18.3 wt%, monocarbonate with 9.2 wt%, hydrotalcite with 2.9 wt%, and calcite with 1.1 wt%. 

### 3.2. Rheological Tests of a Cement Paste with Different Dosages of Superplasticizer

**Figure 4:**
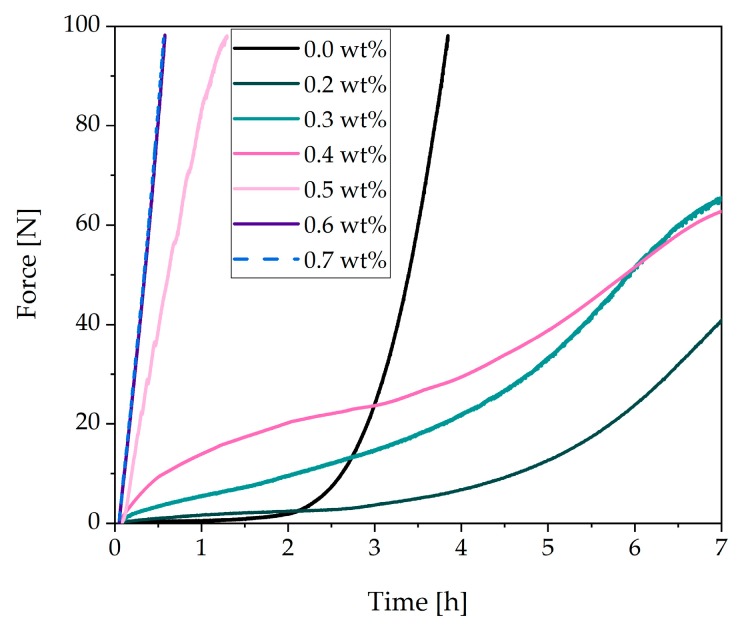
Results of a penetration test (MILD—multipurpose incremental loading device) of CEM I with different dosages of superplasticizer (w/c = 0.36).

In this paper, we aimed to analyze the incompatibility of an SP. This incompatible behavior occurred depending on the dosage. Therefore, different dosages were investigated with the spread flow test (Table 3) and the penetration test (Figure 4). Table 3 shows the spread flow at different dosages (reactive part) 7.5 min after water addition. No sedimentation or bleeding could be observed during the spread flow test. Moreover, the yield stress had been calculated by the equation of Roussel et al. (Equation (3)) [34,35]. The yield stress of the paste decreased significantly at a dosage between 0.2 wt% and 0.5 wt% in comparison to the sample without SP. After a critical dosage of 0.6 wt%, the cement paste did not flow, and the yield stress was very high. The values of the yield stress must be considered with caution because Roussel’s equation does not apply to spread flows < 20 cm.

The results of the penetration test are presented in Figure 4. Without SP, a small increase in the force during the first 2 h could be determined. After 2 h, the force increased rapidly. The penetration test showed retardation of hydration at a dosage of 0.2 wt%. Up to 2 h, the force was comparable to the sample without SP addition. After 2 h, the force needed to penetrate the paste without SP beginning to increase very sharply. This increase could be seen at later points in time in the curve with 0.2 wt% of SP. At a dosage of 0.3 wt% and 0.4 wt%, a constant increase in the curve could be observed. Up to about 3 h, the curves showed higher forces than the sample without SP. During the sharp increase of the sample without SP, the curve passed the others and indicated higher forces. At the critical dosage of 0.5 wt%, the cement paste showed a substantial increase in the force within the first hour and thus a fast loss of flowability at early times. At a dosage of 0.7 wt%, the maximum force of 100 N was reached after 34 min instead of 3.9 h in the system without SP. It was also observed that the samples with high dosages of SP behaved very sensitively. It could be seen in the laboratory that with small changes in the environment, such as the temperature, the paste with a dosage of 0.5 wt% sometimes showed a fast stiffening (penetration test) and sometimes a very liquid behavior (spread flow test). For this reason, a clear overdosage 0.7 wt% was chosen. Therefore, in further study, we concentrated on a dosage of 0.7 wt%.

### 3.3. Analysis of the Incompatible Behavior Caused by Direct Addition of 0.7 wt% of SP

**Figure 5:**
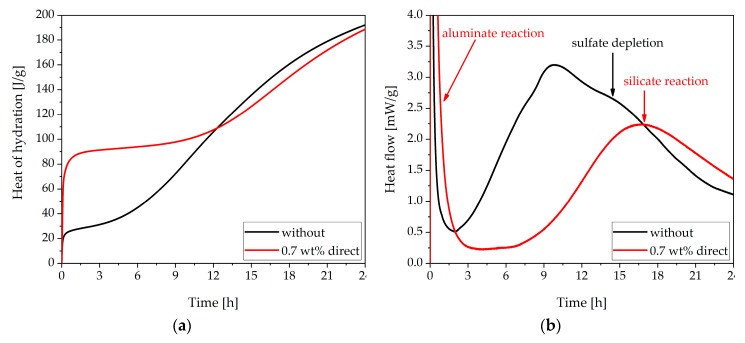
Calorimetric results of CEM I without superplasticizer (SP) and with direct addition of 0.7 wt% of SP (w/c = 0.36): (**a**) Heat of hydration and (**b**) Heat flow.

The incompatible behavior caused by direct addition of 0.7 wt% of SP should be analyzed by calorimetry and in-situ XRD. The results of calorimetric measurements are compared in Figure 5. Both graphs displayed the development of the cement paste sample without SP and of the sample with 0.7 wt% of SP directly added with the mixing water.

In Figure 5a, the heat evolution during the first 24 h of the pure system and the system containing 0.7 wt% of SP are compared. Up to 12 h, the heat of hydration of the sample with SP was much higher than that of the sample without SP. Moreover, the addition of the SP caused retardation of the main period during hydration. As could be observed from Figure 5b, the heat evolution of the second peak (sulfate depletion peak) in the system without SP at 15 h was not very distinct. However, the curve of the sample with SP showed only one peak in the main period. This peak was caused by the retarded silicate reaction. Because of the high heat evolution at the beginning, it could be assumed that the sulfate depletion took place during the initial period, and only the silicate reaction was retarded. In order to prove this assumption, in-situ XRD analysis was performed. Since the aluminate reaction was the process responsible for this problematic behavior, the following XRD analysis focused on phases relating to this reaction.

**Figure 6:**
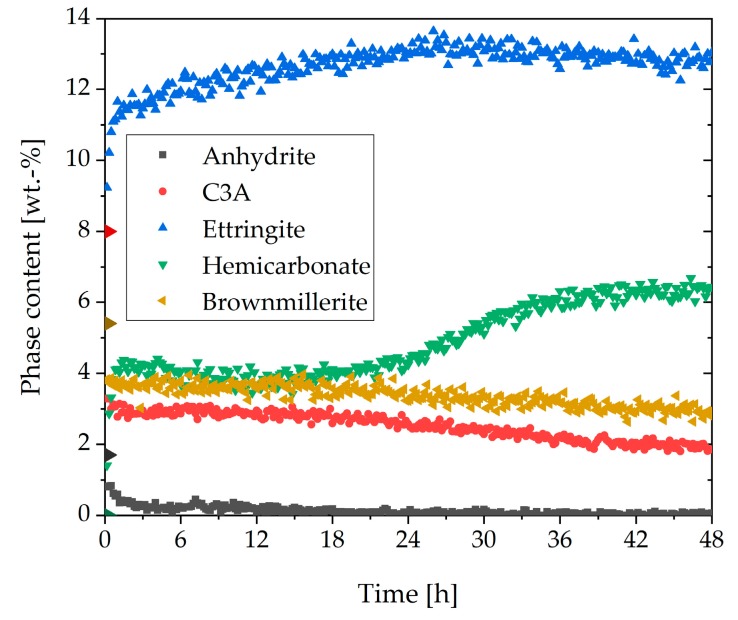
Phase development of the aluminum-containing phases of CEM I with direct addition of 0.7 wt% of SP during 48 h of hydration (w/c = 0.36).

Figure 6 shows the phase content of the sample with 0.7 wt% of SP (direct addition). Bassanite completely dissolved in the first 10 min and was, therefore, not shown. It stands out that the maximum ettringite content of 12.6 wt% was reached nearly at the beginning of hydration and that the anhydrite was consumed rapidly. Moreover, it was remarkable that a considerable amount of hemicarbonate precipitated at the beginning of hydration and increased further after about 25 h. Furthermore, the C_3_A content (3.3 wt%) was much lower than without SP (4.3 wt%) after the initial dissolution. In addition, a considerable amount of brownmillerite was consumed at the beginning of hydration (5.4 wt% to 3.8 wt%), which could not be observed in the system without SP where brownmillerite started to react at a later point in time. After this initial consumption, the amount decreased slowly. After about 22 h, a slow acceleration of the dissolution could be detected.

### 3.4. Two Approaches to Avoiding Incompatible Behavior

In this chapter, two approaches to avoiding incompatible behavior were analyzed. For this purpose, one sample was prepared with an additional amount of 2 wt% of gypsum in order to see the influence of the increased supply of calcium sulfate on hydration. In this case, SP was added directly. Additionally, in the second sample, the SP was added to the cement paste with a delay. Both systems were compared to the system with direct addition of 0.7 wt% of SP (Section 3.3). 

**Figure 7:**
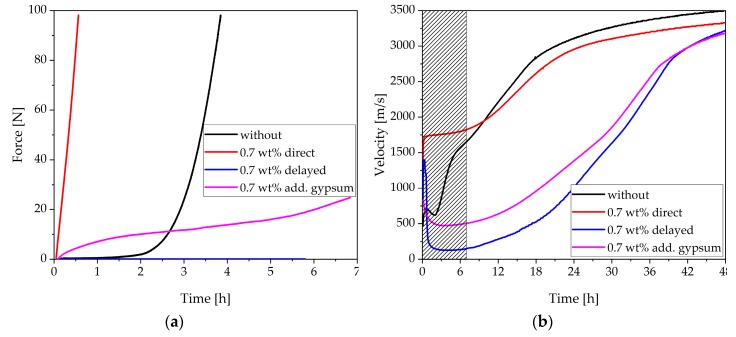
Comparison of the rheological behavior of a sample without SP, with 0.7 wt% of SP (direct addition), 0.7 wt% of SP (delayed addition), and 0.7 wt% of SP (direct addition), and with an additional amount of 2 wt% of gypsum measured with (**a**) a penetration test (MILD), (**b**) ultrasound velocity.

First, the rheological behavior was considered. Figure 7a illustrates the results of the penetration test. The rheological behavior of the sample with a delayed addition had better workability than without SP or with direct addition. The paste with delayed addition and additional gypsum seemed to be more flowable than the system with 0.7 wt% of SP (direct addition), but the system with gypsum showed less flowability than the pure system and the system with delayed addition during the first 3 h of hydration. The paste with a delayed addition showed such a high flowability that no force could be measured until the test is stopped. During the tests, it could be observed that the material with 0.7 wt% of SP (direct addition) and additional gypsum showed a thixotropic behavior. Hours later, the material could still be liquified by appropriate stirring. In order to prove the results of the penetration test, the same samples were measured by ultrasound technique (Figure 7b). The grayed area showed the same hydration time, as seen in Figure 7a. The measured ultrasound velocity was directly influenced by microstructural changes. For this reason, this method could be combined very well with rheological tests. Figure 7b shows that the ultrasound velocity of the sample with 0.7 wt% (direct addition) was very high right from the start. The sample with 0.7 wt% (delayed addition) showed a peak at the beginning. This was caused by a slight water film on the top of the paste, which was not observable in the other systems and, therefore, might not be misinterpreted as a sign of a structure formation (velocity water = 1500 m/s [40]). The results of the sample with a delayed addition showed the lowest ultrasound velocity. The velocity of the sample with gypsum was a little bit higher but much lower than the sample without SP. It could be stated that the results of the penetration test and the ultrasound technique fitted very well. This observation was also made in the literature [44].

**Figure 8:**
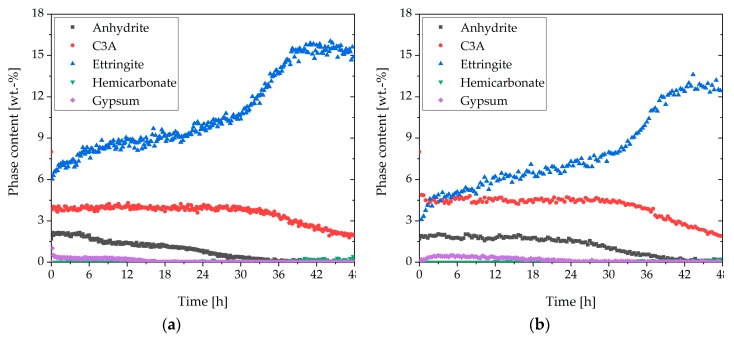
Phase development of the aluminum-containing phases of CEM I during 48 h of hydration (w/c = 0.36) with: (**a**) 2 wt% of gypsum and 0.7 wt% of SP (direct addition), and (**b**) 0.7 wt% of SP (delayed addition).

The observed rheological behavior should be considered in more detail through the analysis of the phase content. Figure 8 shows the in-situ XRD results of the sample with additional gypsum (a) and the direct addition of the SP (b). In contrast to the sample with the direct addition (Figure 6), in Figure 8a, a higher C_3_A content and a lower amount of ettringite are illustrated. In the beginning, the ettringite content reached a value of 6 wt%. Until 30 h, the amount increased steadily. After that, a strong increase could be observed until a plateau was reached (15 wt%). Moreover, no hemicarbonate could be detected. Figure 8b shows the phase content of the sample with 0.7 wt% of SP (delayed addition). The C_3_A content was a little bit higher than in the system with additional gypsum (Figure 8a), and the anhydrite in the system was consumed a little less. The development of the ettringite curve looked comparable to Figure 8a, but the values were lower. In the beginning, the ettringite content was 3 wt%. The curve increased steadily until 35 h. After that, it increased until a plateau of 13 wt% was reached. In these two samples, the bassanite was also completely dissolved in the first 10 min and was, therefore, not shown.

**Figure 9:**
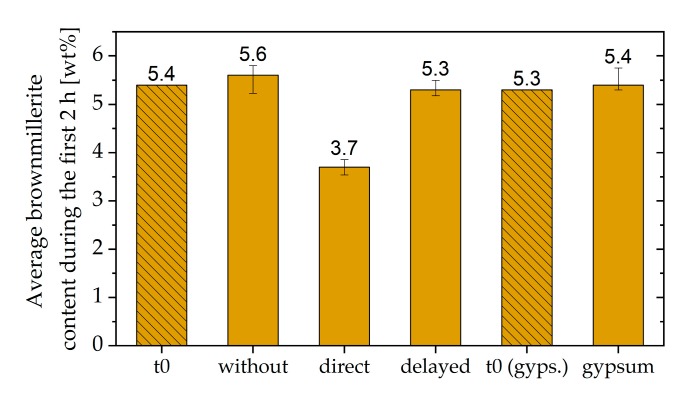
Average brownmillerite content during the first 2 h at the beginning of hydration (t_0_), without SP, with direct addition of 0.7 wt% of SP, with a delayed addition of 0.7 wt% of SP, and with 2 wt% additional gypsum and direct addition of 0.7 wt% of SP.

The average brownmillerite content during the first 2 h of cement hydration was calculated and is plotted in Figure 9. The meaning of t_0_ or t_0 gypsum_ is the amount of brownmillerite at the beginning of hydration (dashed bars). This value was equal in every mixture examined (namely 5.4 wt%), except in the sample with gypsum (5.3 wt%) due to the dilution of the cement by additional gypsum. The error bars represent the lowest and the highest value during the first 2 h. As presented in Figure 9, the content of brownmillerite during the first 2 h was nearly constant for almost all systems. However, as already mentioned in Figure 5, with the direct addition of 0.7 wt% of the SP, significant consumption of brownmillerite could be detected within the first 2 h.

**Figure 10:**
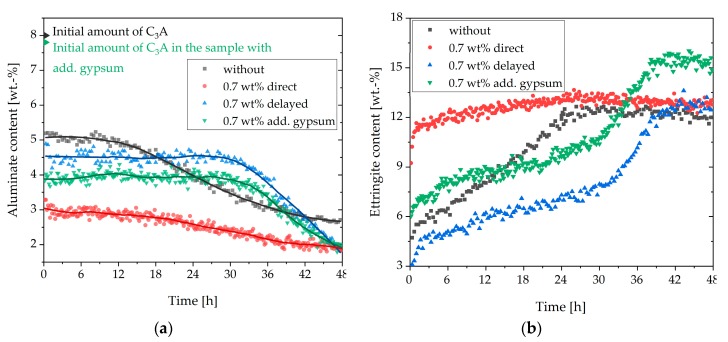
Summary of the (**a**) aluminate and (**b**) ettringite content of the samples without SP, with direct addition of 0.7 wt% of SP, with a delayed addition of 0.7 wt% of SP, with 2 wt% of additional gypsum, and direct addition of 0.7 wt% of SP.

Figure 10a summarizes the C_3_A content of the different samples examined in this study. It could be seen that after the initial fast reaction, the amount of C_3_A in the sample without SP was the highest. In the system with delayed addition of SP, as well as additional gypsum and direct addition, lower C_3_A amounts could be detected, and hence a higher dissolution of C_3_A within the first minutes could be assumed. However, the lowest aluminate content after the initial reaction could be detected in the sample with 0.7 wt% of SP (direct addition). Figure 10b presents the ettringite contents of the different systems. The sample with 0.7 wt% (direct addition) reached the maximum amount of ettringite (12.6 wt%) at the beginning of cement hydration. The sample without SP reached the same amount after 27 h, and the sample with a delayed addition after 39 h. The first two samples decreased slowly after 27 h. The ettringite content of the sample with additional gypsum was lower than the sample with a direct addition until about 33 h. After 33 h, the sample with additional gypsum reached a higher maximum (15 wt%). Until 12 h, the amount of ettringite was higher than the sample without SP. The lowest ettringite content during the illustrated 48 h was shown by the sample with delayed addition. The maximum amount of ettringite was reached after about 39 h.

**Figure 11:**
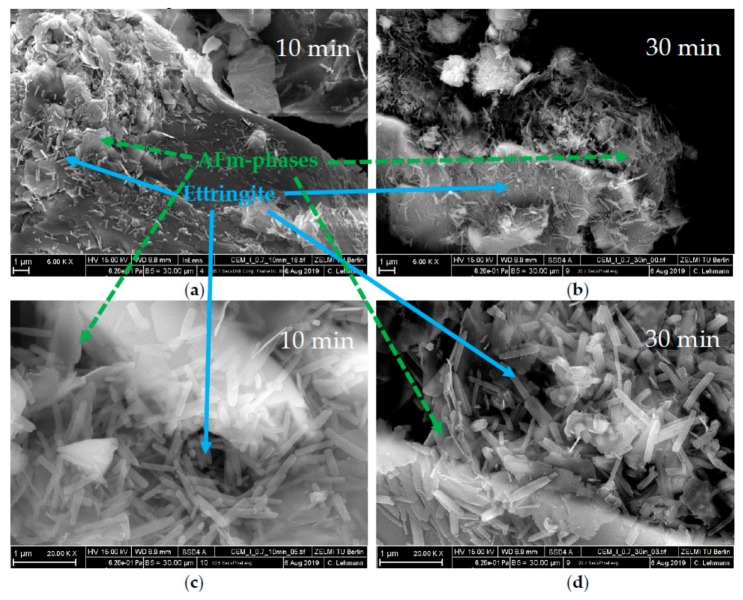
SEM images of CEM I with 0.7 wt% of SP (direct addition) and a w/c ratio of 0.36 after a time of (**a**,**c**) 10 min and (**b**,**d**) 30 min.

**Figure 12:**
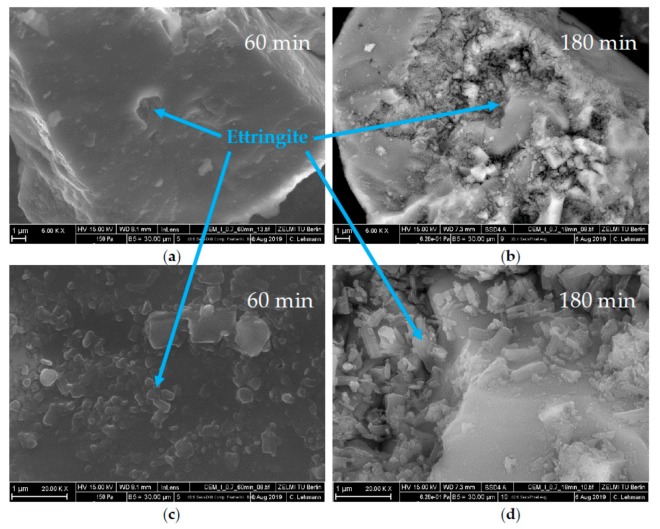
SEM images of CEM I with 0.7 wt% of SP (delayed addition) and a w/c ratio of 0.36 after a time of (**a**,**c**) 60 min and (**b**,**d**) 180 min.

**Figure 13:**
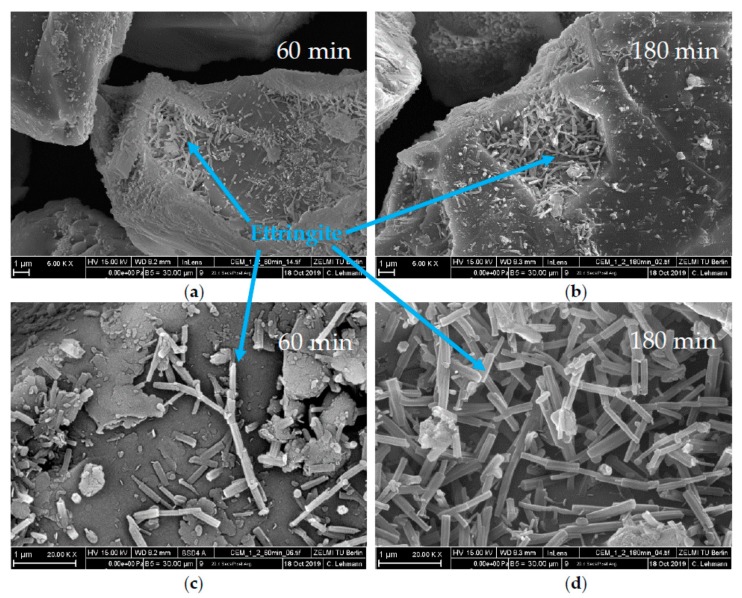
SEM images of CEM I with 0.7 wt% of SP (direct addition), 2 wt% of gypsum, and a w/c ratio of 0.36 after a time of (**a**,**c**) 60 min and (**b**,**d**) 180 min.

SEM images are compared in Figure 11, Figure 12 and Figure 13. For all figures, magnification factors of 5000 ((a) and (b)) and 20,000 ((c) and (d)) were applied, respectively. Figure 11 shows pictures of the OPC used after the direct addition of 0.7 wt% of SP after 10 min (a,c) and 30 min (b,d). A huge amount of ettringite and AFm phases could already be observed after 10 min of hydration. Solely by analyzing the SEM pictures, it could not be concluded which AFm-phase was forming, but the results of the XRD measurements showed that the plate-like structures were hemicarbonate. The content of both phases seemed to increase until 30 min of hydration. Figure 12 shows SEM images after 60 min (a,c) and 180 min (b,d) of the cement paste with a delayed addition of SP. After 60 min, there was still no hemicarbonate observable on the clinker surfaces, and only a few, tiny ettringite crystals could be detected. After 180 min, the amount of ettringite increased, but there were still many free surface areas, and no hemicarbonate was detectable. Figure 13 shows the sample with additional gypsum after 60 min (a) and 180 min (b). No hemicarbonate could be detected. The only crystalline hydrate phase, which could be detected, was ettringite.

## 4. Discussion

The analysis of different SP dosages revealed that the paste stiffened very fast above a critical dosage. The critical dosage was determined by rheological tests, such as the spread flow test (Table 3) and the penetration test (Figure 4). The critical dosage of the stiffening effect starts between 0.5 wt% and 0.6 wt%. For a clear overdosage, 0.7 wt% was chosen for the investigations.

Section 3.3 deals with the question of what causes the incompatibility. For this purpose, the samples without SP and with 0.7 wt% of SP (direct addition) were examined by calorimetry and in-situ XRD.

The heat development during hydration was detected by calorimetry. The sample with 0.7 wt% of SP (direct addition) indicated a sharp increase in the heat of hydration during the first minutes, which was much higher than in the system without SP (Figure 5). In addition, the sample without SP demonstrated two heat flow peaks during the main period. The first peak was caused by the silicate reaction, and the second by the aluminate reaction. The sample with 0.7 wt% of SP (direct addition) only revealed one peak in the main period, which was delayed compared to the main hydration peak of the sample without SP. These two calorimetric figures provided the first conclusion of the incompatibility investigation: when 0.7 wt% of SP was added directly to the cement, the aluminate reaction was accelerated and took place during the initial period. However, the silicate reaction was retarded, as we would expect from the literature [24].

The results of in-situ XRD measurements of the sample without SP (Figure 2) showed that the amount and solubility of the sulfate carrier in the dry cement were appropriate, resulting in corresponding hydration of the OPC with a typical setting time. A steady increase of ettringite and consequently a decrease of C_3_A and anhydrite could be observed during the first day of hydration. The amount of brownmillerite was nearly constant during the first hours. Figure 3 clarifies that ettringite was a metastable phase in the system examined and that after an infinite hydration process of the paste, which was kinetically controlled, the entire amount should be converted into monosulfate and monocarbonate because of the thermodynamic control over the long term. However, it was shown that during hydration of the cement without SP, ettringite was formed and was the only detectable phase from the aluminate reaction until the sulfate was wholly consumed. 

The phase development of the sample with 0.7 wt% of SP (direct addition) (Figure 6) showed precipitation of the maximum ettringite content (12.6 wt%) nearly at the beginning of hydration. Moreover, a considerable amount of hemicarbonate (4.1 wt%) could be detected within the first hour of hydration. The question of what caused the incompatibility could be explained by the formation of ettringite and hemicarbonate. These two phases were responsible for the fast stiffening of the paste. This assumption fitted very well with the investigations of Rößler et al. [8]. According to their study, the formation of AFm reduced the fluidity of a paste. The SEM images of this sample (Figure 11) showed long ettringite crystals. Rößler et al. also figured out that in comparison to short ettringites, long ettringite crystals were related to a loss of flowability [8]. 

It was noticeable that the addition of 0.7 wt% of SP not only accelerated the C_3_A dissolution but also led to an accelerated reaction of brownmillerite within the first hours. Thus, it was concluded that the SP influenced the passivation of both aluminate-containing clinker phases, namely C_3_A and brownmillerite. It seemed that the passivation of C_3_A and brownmillerite occurred later on, or only some parts of the surface were passivated if high amounts of a highly charged SP were present. This assumption arose from the observation that both phases were not entirely consumed during 48 h.

**Figure 14:**
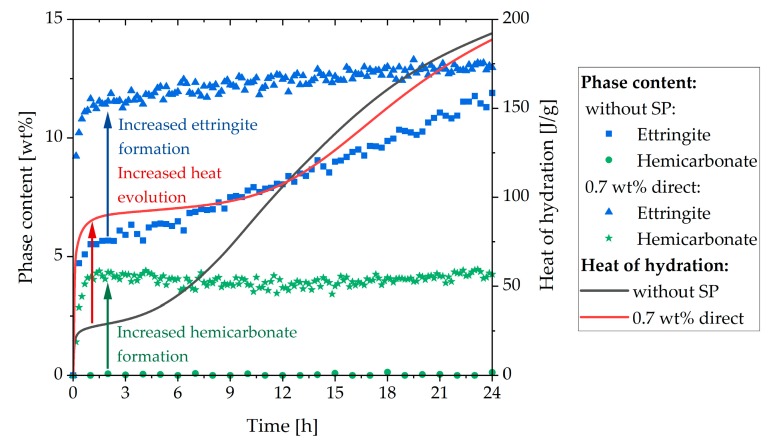
Ettringite and hemicarbonate content of the sample without SP and with direct addition of 0.7 wt% of SP and the heat evolution of both samples.

In summary, the first part of the investigation showed that the incompatibility was caused by an increased formation of ettringite and hemicarbonate. It was shown that the ettringite precipitation was favored in the presence of high dosages of PCE, especially that one with high charge density. This led to faster consumption of sulfates and the earlier appearance of the sulfate depletion peak, which resulted additionally in the formation of hemicarbonate. Due to the rapid water removal from the system caused by the formation of hemicarbonate and, especially ettringite, and the plate-like card house structures of hemicarbonate, a fast loss of fluidity could be observed. Maybe, also, the long-prismatic shape of the ettringite crystals could have an influence, according to Rößler et al. [8]. The passivation of the reactive clinker phase C_3_A and brownmillerite was prevented, and thus a strong ettringite and hemicarbonate formation and a high heat evolution took place in the initial period. These results are summarized in Figure 14.

From the authors’ point of view, there are two possible reasons for the prevention of the initial passivation of the aluminate-containing phases and the resulting rapid stiffening of the sample with direct addition of 0.7 wt% of SP. The first reason could be that the SP first adsorbs at the surface of the aluminate-containing phases and hinders the adsorption of the ions, which are known to passivate the fast reaction of those phases [27,44]. However, this explanation assumes that the adsorbed SP does not passivate the reaction of the aluminate phases. For that reason, some C_3_A can dissolve. The second possibility is that the SP interacts with the ions in the solution and prevents the accumulation on the surface. It has been shown by Myers et al. [44] and Minard et al. [5] that preferably a combination of both calcium and sulfate on the surface of C_3_A is necessary in order to prevent further reaction. Consequently, it should be considered that highly charged SP (negative charge) can interact with calcium ions (positive charge), and the sequestration of calcium ions from the pore solution can lead to a lack of calcium for the passivation of the aluminate phases [27,45]. Especially the adsorbed SP molecules on the surface might lead to a lack of calcium near the surfaces and, as a consequence, inhibit the postulated calcium-sulfate complexes on the aluminate surfaces that inhibit the reaction of the aluminate phases [44].

**Figure 15:**
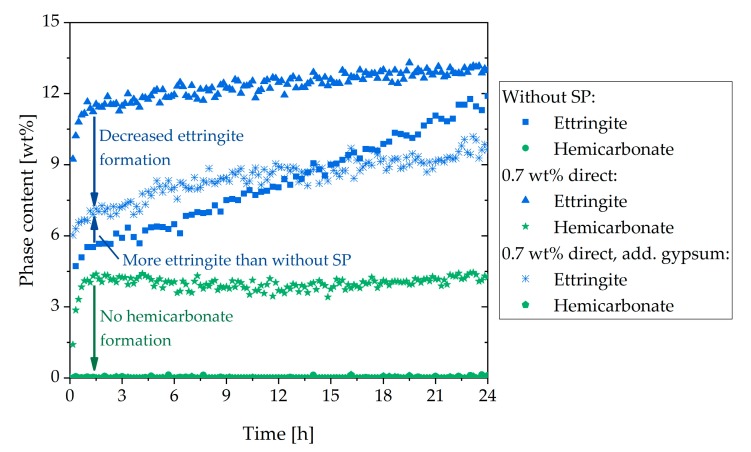
Ettringite and hemicarbonate content of the sample without SP, with direct addition of 0.7 wt% of SP, and with additional gypsum and direct addition of 0.7 wt% of SP.

Section 3.4 deals with the question of how to prevent incompatible behavior. The first approach was the addition of additional gypsum. If further gypsum was added to the dry powder, the undesirable effect did not occur to such a significant extent. The additional amount of gypsum seemed to have two effects. Firstly, the amount of initially reacted C_3_A and C_4_AF (initial period) was significantly reduced compared to the system with direct addition of 0.7 wt% of SP, as shown in Figure 8a, Figure 9, and Figure 10. The additional sulfate might lead to a higher passivation degree compared to the system without additional sulfate. Secondly, no AFm phases were formed (Figure 13) within the first hours of hydration, which were known to have a significant influence on the cement paste fluidity. As a conclusion of the first approach, it could be assumed that the addition of additional gypsum resulted in a less pronounced stiffening caused by less ettringite formation and no AFm phases (Figure 15). However, the incompatible behavior could not be fully prevented due to a continued high ettringite formation.

**Figure 16:**
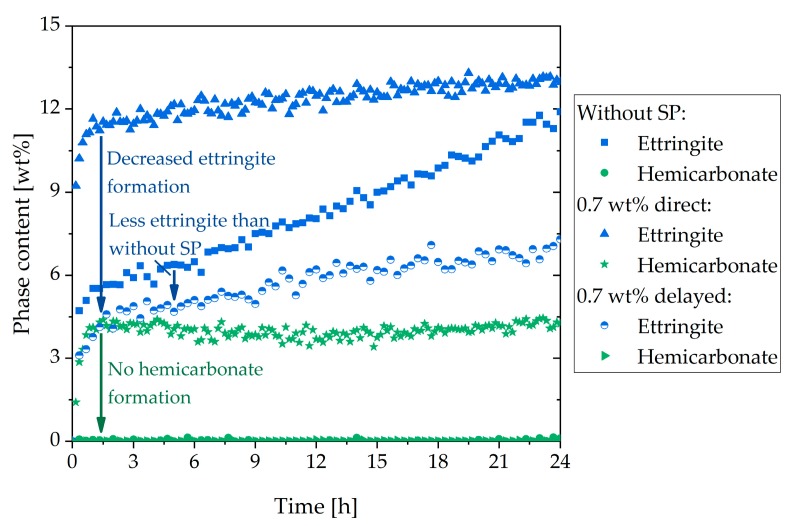
Ettringite and hemicarbonate content of the sample without SP, with direct addition of 0.7 wt% of SP, and with a delayed addition.

The second approach to preventing incompatible behavior was a delayed addition of the SP. It could be observed that the fast ettringite formation did not occur (Figure 16). Quite to the contrary, the amount of formed ettringite was lower than the sample without SP. Thus, fast stiffening could be completely prevented. This was in line with other research [24,30]. If delayed addition of the SP was applied, the passivation of C_3_A could occur before the SP had the chance to interact. Consequently, the undesirable effect did not occur. This was confirmed by the phase development and by the SEM images (Figure 8b and Figure 12). In conclusion, a delayed addition was a better approach to preventing fast stiffening than the addition of additional gypsum.

It should be emphasized that the formation and amount of hydrate phases in the examined systems are responsible for the different rheological behaviors that could be observed in this study. A higher precipitated amount of ettringite observable in the system with an additional gypsum content led to a slight flow loss compared to the plain system without SP. The additional formation of hemicarbonate in the system with 0.7 wt% of SP (direct addition) led to a further flow loss and a fast stiffening in the first minutes of hydration. Hence, the authors would like to raise the point for discussion that not only the amount of the hydrate phase (or solid content in the paste) but also the type of hydrate phase formed (e.g., hemicarbonate instead of ettringite) is crucial to understanding the rheological performance of cement pastes. Additionally, the experiments on the sample with additional gypsum showed that the ettringite that formed stuck together and formed chains if SP was present (Figure 13). Hirsch investigated the influence of the crystallization of pure ettringite in the presence of different types of SP. He stated that polymers with a high anionic charge density had a more significant influence on the crystallization of ettringite than polymers with a low anionic charge density. He found out that polymers mainly influenced longitudinal growth. That is why he inferred that the polymers adsorbed more on the front surface than on the sides [46]. However, since the ettringite chains could only be observed in the sample with gypsum, gypsum must play a crucial role. So far, this phenomenon could not be fully explained and must be further investigated.

## 5. Conclusions

The following conclusions could be drawn from the performed investigations:

- Above a critical dosage, the addition of SP led to a fast stiffening
- This was caused by the formation of vast amounts of ettringite and hemicarbonate
- These two phases were formed because the SP in the initial period prevented the passivation of aluminate-containing phases
- The fast stiffening could be attenuated by adding gypsum and prevented completely by delayed addition of the SP
- Adding gypsum reduced the amount of initially reacted aluminate-containing phases, and no hemicarbonate was formed
- A delayed addition of the SP allowed the passivation of the aluminate-containing phases to develop before the SP was added

The results of this study on the incompatibility of cement and SPs are important for two reasons. Firstly, it is crucial that admixtures are versatile and applicable to different environmental conditions. If SPs are nonrobust, there is only a narrow dosage range for achieving good performance and ensuring that no undesirable loss of flowability, bleeding, or segregation occurs [28]. The second reason why this behavior is interesting is that it is undesirable in this case but could be desirable in others. In 3D printing, for example, a good flowability may be desired during extrusion and a fast stiffening after printing. In that case, the yield stress of the lower layers must be great enough to support the weight of the upper layers [47]. As soon as the investigated behavior is fully understood, and the timing of the rapid stiffening is reliably controllable, this material would offer excellent properties for use in 3D printing.

## Figures and Tables

**Table 1:** Chemical (XRF) and mineralogical composition (XRD) of the CEM I 42.5 R.

Phase	wt%	Phase	wt%	Oxide	wt%	Oxide	wt%
Alite	55.8	Anhydrite	2.2	CaO	64.4	Na_2_O	0.2
Belite	14.6	Bassanite	2.7	SiO_2_	20.4	K_2_O	0.8
C_3_A_cubic_	7.3	Gypsum	-	Al_2_O_3_	5.4	SO_3_	3.1
C_3_A_orth._	3.6	Calcite	3.7	Fe_2_O_3_	2.6	TiO_2_	0.3
Arcanite	0.5	Brownmillerite	7.4	MgO	1.4		

**Table 2:** Chemical data of the superplasticizer.

Solid content (M%)	M_N_^1^ (g/mol)	M_W_^2^ (g/mol)	PDI ^3^	Anionic Charge Density (µeg/g)	Side Chain Length (PEO ^4^)
23	14800	25000	1.69	1530	18

^1^ M_N_ = Number average molecular weight, ^2^ M_W_ = Average molecular weight, ^3^ PDI = Polydisperse index, ^4^ PEO = Polyethylene oxide.

**Table 3:** Spread flow of CEM I with different dosages of superplasticizer (SP) (water/cement, w/c = 0.36) measured by a spread flow test and calculated yield stress (by Roussel).

Dosage of the SP (wt%)	Spread Flow (cm)	Yield Stress (Pa)
0.0	10.75	467.4
0.2	23.50	9.4
0.3	27.00	5.0
0.4	20.75	17.4
0.5	29.75	2.9
0.6	10.00	632.0
0.7	10.00	671.0
